# Planned Combo Strategy for LVAD Implantation in ECMO Patients: A Proof of Concept to Face Right Ventricular Failure

**DOI:** 10.3390/jcm11237062

**Published:** 2022-11-29

**Authors:** Vincenzo Tarzia, Matteo Ponzoni, Demetrio Pittarello, Gino Gerosa

**Affiliations:** 1 Cardiac Surgery and Heart Transplant Unit, Department of Cardiac, Thoracic, Vascular Sciences, and Public Health, University of Padova, 35128 Padova, Italy; 2 Anaesthesia and Intensive Care Unit, University of Padova, 35128 Padova, Italy

**Keywords:** ProtekDuo, RVAD, LVAD, right heart failure, cardiopulmonary bypass

## Abstract

We propose a patient-tailored strategy that considers the risk for postoperative right heart failure, utilizing the percutaneous ProtekDuo cannula (Livanova, London, UK) in an innovative way to perform cardiopulmonary bypass during LVAD implantation in ECMO patients. Our novel protocol is based on the early intra-operative use of the ProtekDuo cannula, adopting the distal lumen as the pulmonary vent and the proximal lumen as the venous inflow cannula during cardiopulmonary bypass. This configuration is rapidly switched to the standard fashion to provide planned postoperative temporary right ventricular support, in selected patients at high risk of right ventricular failure. From September 2020 to June 2022, six patients were supported with the ProtekDuo cannula during and after an intracorporeal LVAD implantation (five of which were minimally invasive): four HeartMate III (Abbott, U.S.A.) and two HVAD (Medtronic Inc, MN). In all cases, the ProtekDuo cannula was correctly positioned and removed without complications after a median period of 8 days. Non-fatal bleeding (bronchial hemorrhage) occurred in one patient (17%) during biventricular support. Thirty-day mortality was 0%. From this preliminary work, our novel strategy demonstrated to be a feasible solution for planned minimally invasive right ventricular support in ECMO patients scheduled for a durable LVAD implantation.

## 1. Introduction

To date, right heart failure (RHF) complicates more than 40% of left ventricular assist device (LVAD) implantations in patients on extracorporeal membrane oxygenation (ECMO) support [1], quadruplicating early mortality [2,3], especially when an unplanned right ventricular assist device (RVAD) is required [4]. The need for a postoperative RVAD affects even long-term prognosis dramatically, both in terms of survival and freedom from RHF recurrence [4]. Moreover, the persistence of RHF 3 months after LVAD implantation has been demonstrated to negatively influence the 2-year survival of patients in the recent analysis of the Society of Thoracic Surgeons/Interagency Registry for Mechanically Assisted Circulatory Support (STS/INTERMACS) Database [5].

Several risk scores for predicting RHF after LVAD implantation have been proposed, but their reliability in discriminating patients with severe manifestations of RHF is still suboptimal [6]. However, even mild to moderate post-implantation RHF can significantly increase the risk of adverse postoperative events, including death [7]. The parameters that are commonly shared by available risk scores usually entail the need for inotropes to sustain the circulation, INTERMACS class I and II, and borderline hemodynamic and echocardiographic measures of right ventricular (RV) performance. Unfortunately, this vulnerable population currently represents almost half of patients who undergo primary LVAD implantation [8]. In addition, being on ECMO support limits the predictive power of echocardiographic measurements, and when fine RV-pulmonary circulation coupling indexes are considered [9]. Thus, anticipating the need for a RVAD after cardiopulmonary bypass (CPB) weaning represents the most significant challenge during LVAD implantation in patients on ECMO support.

In this work, we propose a novel protocol for planning and performing RV support, using a single percutaneous ProtekDuo cannula (Livanova, London, UK), in ECMO patients scheduled for a durable LVAD (d-LVAD) implantation. Our approach provides a unique solution for conducting CPB and for a rapid subsequent switch to a planned temporary RVAD. The preliminary results of the first six patients treated with this strategy are presented.

## 2. Materials and Methods

### 2.1. Population

We retrospectively reviewed all patients on ECMO support scheduled to be upgraded to a d-LVAD, who were implanted using the ProtekDuo cannula at the time of surgery at our Institution between September 2020 and June 2022. The patient’s informed consent was obtained and the institutional Ethics Committee approved the study (protocol 39707, June 2022). The patients’ demographic, clinical, and hemodynamic data were collected from our institutional database of extracorporeal life support (ECLS) and through medical record reviews. Analyzed outcomes were device positioning success, major postoperative complications, and early and late mortality. Follow-up data completeness was 100%.

### 2.2. Protocol

**Figure 1:**
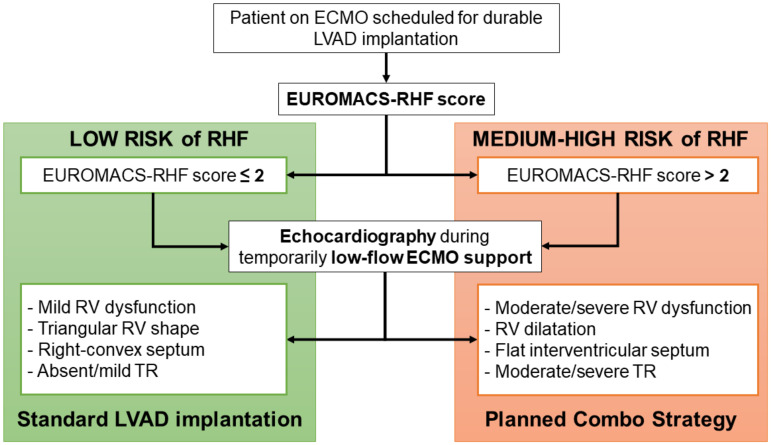
Decision-making algorithm for planning a RV support for ECMO patients scheduled for a durable LVAD implantation. ECMO: extracorporeal membrane oxygenation; EUROMACS-RHF: European Registry for Patients with Mechanical Circulatory Support Right-Sided Heart Failure; LVAD: left ventricular assist device; RHF: right heart failure; RV: right ventricle; TR: tricuspid regurgitation.

Patients on ECMO support who were planned to be upgraded to a d-LVAD underwent a clinical and echocardiographic evaluation to assess the risk of post-implantation RHF (Figure 1).

**Figure 2:**
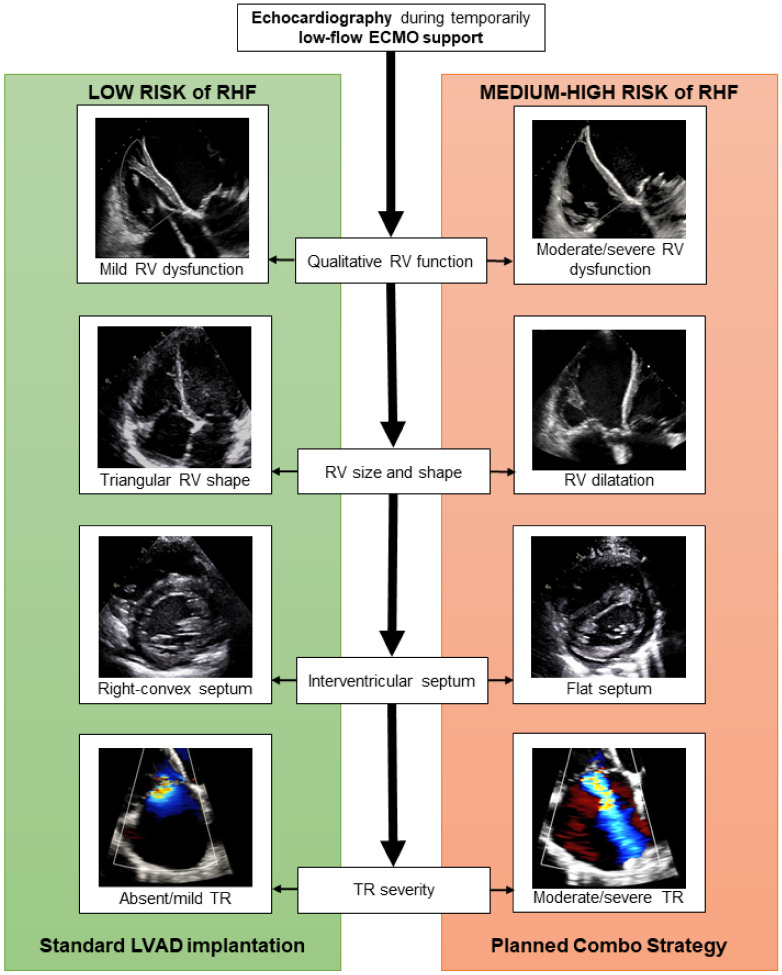
Echocardiographic parameters of RV performance that guide d-LVAD implantation strategy in ECMO patients. RV: right ventricle; TR: tricuspid regurgitation.

Firstly, the EUROMACS (European Registry for Patients with Mechanical Circulatory Support) Right-Sided Heart Failure Risk Score was calculated [10], to initially distinguish between low risk (score ≤2) and medium–high risk (score >2) of RHF. Since the reliability of this score has been demonstrated to improve when associated with a more detailed evaluation of RV performance [6], we meticulously assessed RV ventricular function, shape, and dimensions through echocardiography during temporarily reduced ECMO support. Figure 2 shows whether exemplificative echocardiographic images of RV function support the use of the *Planned Combo Strategy*.

### 2.3. Surgical Technique

**Figure 3:**
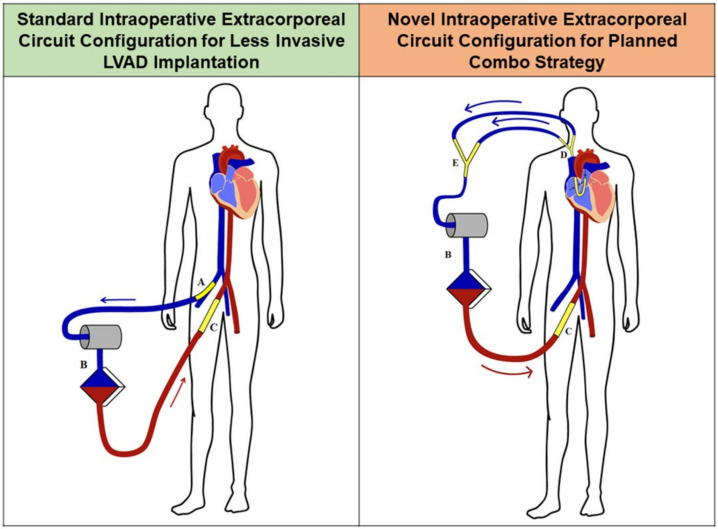
Schematic representation of the standard intraoperative circuit for CPB during less invasive d-LVAD implantation (**left panel**) and the novel configuration during the Planned Combo Strategy where the ProtekDuo cannula was used as venous inflow and pulmonary vent (**right panel**). A: Femoral venous line; B: CPB machine; C: femoral arterial line; D: ProtekDuo cannula; E: Y connector on the venous line.

Under fluoroscopy and transesophageal echocardiographic guidance, the ProtekDuo cannula was inserted percutaneously in the right jugular vein and advanced across the pulmonary valve, as described elsewhere [11,12,13]. The arterial cannula of the previous ECMO was connected to the CPB circuit, which was initiated using the proximal lumen of the ProtekDuo cannula as venous inflow and the distal lumen as a pulmonary vent, through a Y connection on the venous line (Figure 3). The venous cannula of the previous ECMO was then removed. To reduce the need for complete heparinization, the hemodynamic support during the procedure could even be provided using the ECMO circuit itself, avoiding CPB and full heparinization. In this setting, both lumens of the ProtekDuo cannula (using a Y connector) were connected to the venous line of the ECMO circuit, through a rapid switch. The arterial cannula of the ECMO was left in place and the previous venous cannula was removed. Circulatory and respiratory support during the LVAD implantation was thus provided with this minor modification only of the ECMO circuit on the venous side.

Intracorporeal LVAD (i-LVAD) implantation was performed with a sternal-sparing technique preferentially. Through a left mini-thoracotomy in the fifth intercostal space, the HVAD (HeartWare, Medtronic, Minneapolis, MN, USA) or Heartmate III (Abbott, Abbott Park, IL, USA) device was implanted on the beating heart on CPB/ECMO. A right anterior mini-thoracotomy was performed in the second intercostal space and the outflow graft was anastomosed to the ascending aorta [14]. The LVAD support was started and the patient was gently weaned from CPB/ECMO. Subsequently, the ProtekDuo cannula was rapidly connected to a magnetically levitated centrifugal pump (Levitronix CentriMag, Levitronix LLC, Waltham, MA, USA) for RV support, using the proximal lumen as the inflow line and the distal lumen as the outflow line, such as in the standard fashion [11,12,13]. The LVAD and RVAD supports were then balanced to achieve optimal cardiac output.

### 2.4. Statistical Analysis

The data were summarized as the mean (standard deviation [SD]) or median (interquartile range [IQR]) for quantitative variables, as counts and percentages for categorical variables. Survival was estimated with the Kaplan–Meier method. Analyses were performed using SPSS 23.0 (IBM Corporation, Armonk, NY, USA).

## 3. Results

In the study period, we used the ProtekDuo Cannula during and after 10 LVAD implantations in ECMO patients. Among these cases, six patients (mean age 56 [13] years, 100% male) underwent d-LVAD implantation from ECMO using the ProtekDuo cannula intraoperatively and then postoperatively to provide RV support, as a *Planned Combo Strategy*. The other four patients (mean age 53 [11] years, 75% male) developed an unexpected RHF after three extracorporeal and one i-LVAD implantations, requiring an unplanned RVAD. We herein present the results of the new Planned Combo Strategy.

Left heart failure etiology was ischemic dilated cardiomyopathy in three patients (50%) and primitive dilated cardiomyopathy in three (50%). In two cases (33%), the patient had already undergone a cardiac surgical procedure (aortic valve and ascending aorta replacement in one and single coronary artery bypass graft and mitral valve repair in the other). The mean EUROMACS-RHF score was 4.9 (1.2) and in all cases the patient had at least one criterion of impaired RV performance at echocardiography. Demographic and preoperative clinical characteristics are summarized in Table 1.

Four patients (67%) were implanted with HeartMate III and two (33%) with HVAD. In five of six implantations, a minimally invasive technique was adopted: bi-thoracotomy in four cases and left anterior mini-thoracotomy + mini-sternotomy in one (Table 2).

In all cases, the ProtekDuo cannula was correctly positioned without complications and it provided satisfactory RV support (mean maximal flow of 4.2 [0.6] L/min) for a median of 8 (4–16) days. In one case (17%), an oxygenator was included in the RVAD circuit for respiratory support while the pulmonary function was recovering from cardiogenic pulmonary edema. During RVAD support, major bleeding occurred in one patient (17%): a bronchial bleeding requiring prolonged mechanical invasive ventilation and bronchial toilettes. The ProtekDuo cannula was easily removed at bedside without complications in all cases. During hospitalization, other major complications were: the need for temporary tracheostomy in three cases (50%), new-onset acute renal injury requiring renal replacement therapy in two cases (33%), sepsis in one case (17%), and major ventricular arrhythmias in one case (17%, see Table 2). Overall 30-day and 90-day mortality was 0% (0/6) and 17% (1/6), respectively. The patient who expired had developed septic shock and multi-organ failure 58 days after RVAD removal. None of the patients developed RHF after RVAD discontinuation.

## 4. Discussion

The upgrade from a temporary ECMO support to a d-LVAD in patients with cardiogenic shock requires a meticulous clinical evaluation since the outcomes in this challenging population are historically expected to be poor, especially when multiple risk factors are present [1,15,16]. One of the most important determinants of the patient’s prognosis is the occurrence of RHF [1,2,3], which can significantly increase early and long-term mortality, even in mild-to-moderate forms [7]. Therefore, it is not surprising that the unexpected need for RVAD after LVAD implantation almost halves the chances of survival [4]. In the present study, we hypothesized that the early adoption of a planned RV mechanical support in selected patients at high risk of post-implantation RHF could represent a promising strategy for ECMO patients scheduled to have d-LVADs. Moreover, we opted for the utilization of the percutaneous ProtekDuo cannula to provide minimally invasive intra- and postoperative RV support.

The actual scores designed for predicting the risk of RHF after LVAD implantation are still limited by suboptimal reliability. Sert et al., specifically compared the predictive power of available scores and found that the EUROMACS-RHF score displayed the highest discrimination power in their cohort [6]. Moreover, the authors demonstrated that the specificity and sensibility of the RHF scores can be enhanced by combining them with other hemodynamic and echocardiographic parameters [6]. However, by unloading the RV, the ECMO support alters RV preload and its measured performance, impeding an accurate evaluation of RV function [9]. For these reasons, we designed an institutional protocol for ECMO patients scheduled for d-LVAD implantation that considers both the EUROMACS-RHF score and a qualitative echocardiographic evaluation of the RV during a temporary low-flow ECMO support (Figure 1 and Figure 2). When a moderate-to-high risk of RHF was present, a Planned Combo Strategy was adopted. This approach provides an early and planned instauration of RVAD support from CPB weaning, which is accomplished by using the ProtekDuo cannula as a part of the CPB circuit itself.

Traditional techniques for RV support require a sternotomy or a thoracotomy, adding further invasiveness to the LVAD implantation procedure, limiting early mobilization of the patient, and increasing the sources of bleeding and infection [1,2,5]. In addition, when RV support is no longer necessary, the removal of the device requires surgical re-entry, or, in the case of a tunneled prosthetic graft, it entails prolonged retention of foreign material in-situ [17]. Recently, the ProtekDuo cannula has emerged as an attractive option for RV support in patients with left ventricular failure developing temporary RHF in different clinical settings (see Table 3) [11,12,13,18,19,20]. This double-lumen cannula offers all the well-known advantages of a totally percutaneous approach, together with the capability to provide >4 L per minute of blood flow. We routinely adopted a third-generation magnetically levitated continuous flow pump, with proven hemocompatibility and safety for medium-term support [21], and implementability with an oxygenator.

Due to its favorable characteristics of safeness and efficacy, the ProtekDuo cannula has been adopted to treat the occurrence of RHF after LVAD implantation mainly as an unplanned strategy [11,12,13]. Since a planned instauration of right mechanical support after LVAD implantation has demonstrated clear advantages [3,4], we hypothesized an intraoperative adoption of the ProtekDuo cannula in selected patients at moderate-high risk of RHF: the *Planned Combo Strategy*. Utilizing the ProtekDuo cannula since the establishment of CPB and subsequently providing postoperative RV mechanical support in a planned manner has several potential advantages. Firstly, this strategy offers a total peripheral CPB, upgraded with a pulmonary decompressing vent (Figure 3), which is extremely helpful in minimally invasive LVAD implantations and in reinterventions. In ECMO patients, intraoperative mechanical support can even be provided by the ECMO itself, without transitioning to CPB, which was recently proved to shorten operative times and reduce the risk of bleeding [22]. This intraoperative support can be rapidly switched to a temporary postoperative RVAD. Biventricular support is thus initiated, reducing the need for inotropes to sustain the hemodynamics after the LVAD implantation, ensuring a physiological circulation across the lungs and full-flow support, which we have shown to ameliorate patients’ outcomes with respect to the standard ECMO cannulation [23]. If combined with a sternal-sparing LVAD implantation, the ProtekDuo cannula allows extremely rapid patient extubation and mobilization, with improved early outcomes [24,25,26]. Moreover, in the case of pulmonary edema, an oxygenator can be included in the RVAD circuit, until pulmonary function recovers.

During biventricular support, major bleedings may affect 20–40% of patients [27,28] and our preliminary findings showed a 17% rate of bleedings (non-fatal bronchial hemorrhage) that required intervention during RVAD + LVAD support. On the contrary, thrombotic events never occurred during biventricular support in our experience. As demonstrated in isolated LVAD implantation [24,29], we speculate that a minimally invasive approach may further reduce the rates of these complications, although further data are needed in the setting of the Planned Combo Strategy.

During RVAD support, RV performance is monitored constantly via seriate echocardiography. By progressively reducing the RVAD flow, the RV is allowed to gently preload, potentially reducing the risk of unexpected RHF. When RV recovery occurs, satisfying device weaning criteria [11,12], decannulation can be accomplished bedside, with only one single deep hemostatic stitch. We suggest a careful post-removal echocardiographic assessment, since novel severe tricuspid regurgitation may affect up to one-third of patients [11], although this complication never occurred in our small series. As shown by our echocardiographic data after RVAD removal (Table 2), RV function usually improves, as well as the severity of tricuspid valve regurgitation in most cases.

Early mortality of ECMO patients with LVAD implantation can be as high as 24–27% [1,22,30]. We have previously shown that in-hospital mortality following ECLS instauration ranges from 20 to 47%, depending on the acute vs. chronic etiology of heart failure [16], the percentage of flow required to sustain the circulation [23], and the type of device adopted (ECMO vs. paracorporeal LVAD) [23]. In addition, having a high risk of RHF further negatively affects the patient’s prognosis [2,3,4,5], contributing to the creation of an extremely challenging setting for d-LVAD implantation. In the present preliminary series, 30-day and 90-day mortalities were 0% (0/6) and 17% (1/6), respectively. In other words, none of the patients developed RHF after RVAD removal and the only death was not cardiovascular-related. These findings align with previous reports in which a planned RVAD support with the ProtekDuo cannula was scheduled for patients undergoing d-LVAD implantation (Table 3). Noticeably, weaning rates from the RVAD and patients’ outcomes seem to be inferior when the ProtekDuo cannula is used as a rescue strategy if postoperative RV failure occurs [13]. Our preliminary work aims to stimulate a prospective collection of patients to support these findings on a larger scale.

## 5. Limitations

Being composed of a small number of treated patients with a short-term follow-up, the present study represents a preliminary evaluation of the feasibility of our new Planned Combo Strategy and we consider it as a proof of concept of its technical practicability and reproducibility. The limited sample size, the lack of patients’ demographic and clinical diversity, and the short-term follow-up must dictate particular caution in the interpretation and generalization of our early findings. Unfortunately, the lack of an adequate control group does not allow us to define the efficacy, efficiency, and safety of this strategy, which, however, go beyond the aims of the present work. A prospective and controlled enrolment of a larger number of patients is paramount for this purpose.

## 6. Conclusions

Planning the need for RV support in ECMO patients who are scheduled for a d-LVAD implantation is mandatory. We herein present a novel patient-tailored approach, the *Planned Combo Strategy*, that considers the risk for postoperative RHF, together with its preliminary results on the first six treated patients. Adopting the ProtekDuo cannula, we provide a unique solution for conducting CPB, utilizing the distal lumen as a pulmonary vent to decompress the left ventricle and the proximal lumen as the venous inflow line, and for a rapid subsequent switch to a temporary RVAD. This technique, if combined with a sternal-sparing LVAD implantation, permits true minimally invasive biventricular support. In our limited series, this strategy was found to be feasible with encouraging rates of morbidity and mortality, during a median RV support of 8 days. Further studies are encouraged to prove its efficacy and safety on a larger scale.

## Figures and Tables

**Table 1:** Demographic characteristics and preoperative clinical and echocardiographic details of patients (*n* = 6). ECMO: extracorporeal membrane oxygenation; EUROMACS: European Registry for Patients with Mechanical Circulatory Support; INTERMACS: Interagency Registry for Mechanically Assisted Circulatory Support; IQR: interquartile range; SD: standard deviation.

	Mean (SD)	Median (IQR)
Age (years)	56 (13)	61 (42–66)
Body surface area (m^2^)	1.8 (0.2)	1.8 (1.6–2.1)
EUROMACS score for RHF	4.2 (0.6)	4.3 (3.6–4.7)
Cardiac Index (L/min/m^2^)	2.1 (0.2)	2.2 (2–2.3)
Left ventricular ejection fraction (%)	20 (5)	18 (17–26)
Right ventricular fractional area change (%)	20 (11)	21 (11–29)
Left ventricular end-diastolic volume (mL/m^2^)	175 (31)	186 (150–195)
Right ventricular end-diastolic area (cm^2^/m^2^)	18 (3)	18 (15–21)
Systolic pulmonary artery pressure (mmHg)	45 (15)	45 (30–59)
Number of inotropes	2.5 (0.5)	2.5 (2–3)
Creatinine (mg/dL)	1.8 (0.3)	1.7 (1.5–1.9)
Hemoglobin (g/L)	10.5 (1.7)	11 (9.1–12.3)
Platelet count (103/μL)	205 (15)	210 (190–225)
	N	%
Male	6	100
Left heart failure etiology		
Ischemic dilated cardiomyopathy	3	50
Primitive dilated cardiomyopathy	3	50
Preoperative ECMO	6	100
Previous cardiac surgery	2	33
Previous percutaneous coronary interventions	3	50
Mechanical invasive ventilation	2	33
Renal replacement therapy	2	33
INTERMACS class		
Class I	6	100
Mitral valve regurgitation grade		
Mild	2	33
Moderate	2	33
Severe	2	33
Tricuspid valve regurgitation grade		
Absent	1	17
Mild	2	33
Moderate	3	50
Flattening of intraventricular septum at echocardiography	3	50
Qualitative right ventricular performance		
Mildly impaired	1	17
Severely impaired	5	83

**Table 2:** Intraoperative course and clinical and echocardiographic postoperative details of patients (*n* = 6). ECMO: extra-corporeal membrane oxygenation; IQR: interquartile range; LVAD: left ventricular assist device; RVAD: right ventricular assist device; SD: standard deviation.

	Mean (SD)	Median (IQR)
RVAD support period (days)	10 (8)	8 (4–16)
RVAD maximal flow (L/min)	4.2 (0.6)	4.3 (3.6–4.7)
Mechanical ventilatory support (days)	10 (8)	7 (4–20)
Intensive care unit stay (days)	31 (30)	23 (12–41)
Right ventricular fractional area change (%) after RVAD removal	28 (3)	30 (25–31)
Right ventricular end-diastolic area (cm^2^/m^2^) after RVAD removal	13.3 (2)	12.8 (11.7–15.3)
	N	%
ECMO circuit to perform cardiopulmonary bypass	2	33
Durable LVAD type		
Heartmate III	4	66
HVAD	2	33
LVAD implantation technique		
Bi-thoracotomy	4	66
Left thoracotomy + mini-sternotomy	1	17
Full sternotomy	1	17
Oxygenator in RVAD circuit	1	17
ProtekDuo cannula positioning success	6	100
Major bleeding during RVAD support	1	17
Thrombosis during RVAD support	0	
Postoperative complications during hospitalization		
Tracheostomy	3	50
New-onset acute kidney injury	2	33
New-onset renal replacement therapy	2	33
Sepsis	1	17
Ventricular arrhythmias	1	17
Mitral valve regurgitation grade after RVAD removal		
Absent	4	66
Mild	2	33
Tricuspid valve regurgitation grade after RVAD removal		
Absent	1	17
Mild	3	50
Moderate	2	33
Flattening of intraventricular septum after RVAD removal	1	17
Qualitative right ventricular performance after RVAD removal		
Mildly impaired	3	50
Moderately impaired	2	33
Severely impaired	1	17
30-day mortality	0	
90-day mortality	1	17

**Table 3:** Literature review: ProtekDuo cannula used for RV support in patients with left ventricular failure developing temporary RHF in different clinical settings. LVAD: left ventricular assist device; RVAD: right ventricular assist device.

Author	Patients	Treatment of Left Ventricular Failure	Timing of Implantation of ProtekDuo	RVAD Support Duration	Outcomes
Salna et al. [11]	27	Durable intracorporeal LVAD	After LVAD implantation	Median: 11 (7–16) days	Weaning rate from RVAD: 86%. Need for durable RVAD: 11%. In-hospital mortality: 15%. Complications rate: 57%.
Vijayakumar et al. [12]	1	Heartware HVAD	After LVAD implantation	36 days	The patient was weaned from RVAD without complications during support.
Ravichandran et al. [13]	17	Durable LVAD (12 pts), heart transplantation (2 pts), and TandemHeart (1 pt)	After LVAD implantation or heart transplantation	Mean: 10.5 ± 6.5 days	Weaning rate from RVAD: 23%. Need for durable RVAD: 35%. Mortality on RVAD: 41%. Complications rate: 35%.
Carrozzini et al. [18]	3	Heart transplantation	After heart transplantation	4, 9, and 12 days	All patients were weaned from RVAD and discharged home. Internal jugular vein thrombosis occurred in 1 patient.
Ruhparwar et al. [19]	2	Impella 5.0/5.5	Concomitant to Impella implantation	20 and 31 days	Both patients were bridged to durable LVAD implantation without RVAD-related complications.
Schmack et al. [20]	11	Heartware HVAD (6 pts) and HeartMate III (5 pts)	Concomitant to LVAD implantation	Mean: 16.8 ± 9.5 days	30-day survival: 72.7%. Weaning rate from RVAD: 90.9%. No severe RVAD-related complications.
**Present Study**	6	Heartware HVAD (2 pts) and HeartMate III (4 pts)	Concomitant to LVAD implantation	Median: 8 (4–16) days	Weaning rate from RVAD: 100%. 30-day survival: 100%. 90-day survival: 83%. RVAD-related complications rate: 17%.

## Data Availability

Data available on request to the corresponding author.
